# Corticospinal Excitability and Inhibition Are Not Different between Concussed Males and Females

**DOI:** 10.3390/brainsci12070824

**Published:** 2022-06-24

**Authors:** Alexandra Pauhl, Alia Yasen, Anita Christie

**Affiliations:** 1 Faculty of Health Sciences, School of Kinesiology, Western University, London, ON N6A 2X2, Canada; apauhl@uwo.ca; 2 Department of Human Physiology, University of Oregon, Eugene, OR 97403, USA; alia.yasen@gmail.com

**Keywords:** sex differences, corticospinal excitability, corticospinal inhibition, concussion, TMS

## Abstract

It has been consistently demonstrated that females report greater numbers of concussions in sex-comparable sports and take longer to recover from concussive symptoms than males. However, it is unknown if the neurophysiological consequences of concussion may contribute to these sex differences in concussion symptoms and recovery. The purpose of this study was to examine potential sex-related differences in neurophysiology in healthy and concussed individuals. Twenty-one (nine F) concussed individuals (20.9 ± 4.1 years; CONC) and twenty-one age-, sex-, height-, weight-, and activity-matched controls (21.2 ± 4.2 years; CONT) participated in the study. The CONC group reported to the lab within 72 h, 1-week, and 2-weeks post-injury and the CONT group followed a similar measurement schedule. Using transcranial magnetic stimulation, motor evoked potential (MEP) amplitude and cortical silent period (CSP) duration were measured from the first dorsal interosseous muscle to assess corticospinal excitability and inhibition, respectively. There were no significant differences across time (*p* ≥ 0.13) or between the CONC and CONT group in MEP amplitude (*p* = 0.72) or CSP duration (*p* = 0.54). Overall, males (119.08 ± 29.91 ms) had significantly longer CSP durations compared with females (101.24 ± 33.43 ms), indicating greater corticospinal inhibition in males, regardless of injury status (*p* = 0.04). An important and novel finding of this study was the lack of differences in these neurophysiological measures between males and females following concussion. To our knowledge, this is the first study to document greater corticospinal inhibition in males compared with females.

## 1. Introduction

Concussion is a major health problem in children and young adults, as these groups are highly represented in sport-related head injuries [1]. A concussion is a sub-category of mild traumatic brain injury (mTBI), which represents 70% to 90% of all treated TBIs [2]. A harmful neurometabolic cascade can occur due to the biomechanical insult of concussion. This cascade can result in a metabolic mismatch between the increasing energy demand and decreasing supply, and an excitatory-inhibitory neurotransmitter imbalance [3,4,5]. In humans, post-injury recovery can be observed and measured through an acute stage (24–72 h), sub-acute stage (4 to ~14 days), and for some individuals, a persistent symptom or chronic stage (>10–14 days for adults, >four weeks for children) [6].

Glutamate and gamma aminobutyric acid (GABA) are the primary excitatory and inhibitory neurotransmitters, respectively, that maintain the excitatory-inhibitory balance within neurons in the brain [7]. This neurotransmitter balance is required for maintaining typical neurological function. Therefore, an excitatory-inhibitory imbalance post-concussion could have multiple consequences such as greater susceptibility to a subsequent and more severe concussion, memory and attention dysfunction, and prolonged symptoms [8,9,10]. Magnetic resonance spectroscopy (MRS) research has demonstrated that this neurotransmitter imbalance may occur during the sub-acute post-injury stage and continue into the chronic post-injury stage. For example, Yasen et al. (2018) observed that the glutamate to GABA ratios in the dorsolateral prefrontal cortex were significantly higher at two weeks post-injury, representing an excitatory-inhibitory imbalance [11]. Using similar MRS techniques, Tremblay and colleagues also suggested that there may be an excitatory-inhibitory imbalance in the chronic post-injury stage [10]. These findings support the notion that even if post-concussion patients are asymptomatic, their brains can still have an altered neurometabolism that does not return to pre-injury state [12].

Evidence from previous studies using transcranial magnetic stimulation (TMS) has shown alterations in corticospinal excitability and inhibition, possibly due to the excitatory-inhibitory neurotransmitter imbalance [13]. Although other measures of corticomotor excitability exist, in concussed individuals, MEP amplitude is the most widely studied, and other measures, such as intracortical facilitation, have been found to be similar between control and concussed participants [14]. Using motor evoked potential (MEP) amplitude to determine corticospinal excitability, studies have demonstrated mixed findings. Lower excitability has been reported in individuals with mTBI [15], but other studies either found no significant differences in MEP amplitude between an acute mTBI and control group [13,14,15,16,17,18,19], or found that a concussion group generally had greater excitability [20]. These differences across studies may reflect differences in post-injury testing time, as in animal models, excitability changes within minutes to hours post-injury [4].

Findings for changes in corticospinal inhibition, using the cortical silent period (CSP) duration, are more consistent across studies. Studies using paired-pulse techniques to assess short- and long-interval intracortical inhibition consistently show no differences between control and concussed individuals [14,21,22,23]. Although there are some studies with participants in the chronic post-injury stage reporting no differences in CSP duration compared with controls [13,24], the CSP duration is typically reported to be longer among concussed individuals, indicating greater inhibition in concussed individuals in the acute and sub-acute post-injury stages. Numerous studies have observed that CSP duration is longer at 72 h post-injury and remains longer up to two months post-injury [16,20,25], as well as nine months and 30 years post-injury [10,14,21]. These longer CSP durations have been associated with slower movement speeds and reaction times [21], which may explain the motor deficits observed post-injury [26,27,28]. Although the majority of these studies included both male and female participants, potential sex-based differences in the neurophysiology were not examined.

It has been consistently demonstrated that females generally report a greater number of concussions than males in sex-comparable sports [29] and take longer to recover from concussion than males [30,31,32,33]. Longer recovery in females is evidenced by females generally reporting greater post-concussion symptom (PCS) scores and being more likely to miss more than seven days of normal activities due to concussive symptoms [30]. It has also been observed that females are at a greater risk of experiencing persistent post-concussion symptoms, as they have demonstrated greater mean PCS scores at three-months post-injury compared with males [31,33]. Although the reasons for these sex-based differences are not yet fully understood, numerous factors have been suggested to contribute, including: honesty in reporting symptoms, different hormonal systems, and head and neck musculature. Neural physiology and cellular responses [34] have also been suggested as potential contributors to sex-related differences in PCS. However, it is unknown if the neurophysiological consequences of concussion described above differ across sexes.

Therefore, the purpose of this study was to determine sex-based differences in neurophysiology in the acute and sub-acute post-injury stages. Corticospinal excitability and inhibition were assessed across a two-week period in concussed (CONC) and healthy control (CONT) males and females. It was hypothesized that: (i) corticospinal excitability would be similar between groups and sexes, and (ii) corticospinal inhibition would be greater in the CONC group compared to the CONT group, but similar between sexes.

## 2. Materials and Methods

### 2.1. Participants

Participant data collection was conducted as part of a study in the Neurophysiology Laboratory at the University of Oregon. A total of 48 participants were included in this study, with 24 participants (12 males and 12 females) in each of the CONC and CONT groups. Individuals in the CONC group were diagnosed by a specialized health professional (certified athletic therapist or physician). Individuals in the CONT group were sex-, age-, height-, weight-, and activity-matched to each participant in the CONC group.

All participants provided written informed consent and were asked to complete a brief medical history and TMS safety screening questionnaire [35]. Exclusion criteria for all participants included: (i) a history of two or more concussions or a concussion (in addition to the current one for the CONC group) within a year prior to testing; (ii) history of cognitive deficiencies such as memory loss or difficulty concentrating (unrelated to concussion); (iii) history of attention deficit hyperactivity disorder, neurological impairments, musculoskeletal impairments, or seizures; (iv) use of medications that may impact TMS-based measures or neuromuscular function (e.g., anti-depressants, anti-seizure medication); or (v) contraindications to the use of TMS. Additional exclusion criteria specifically for the CONC group included loss of consciousness for more than one minute. All procedures were reviewed and approved by the Institutional Review Board of the University of Oregon.

### 2.2. Measurements

Each participant completed three testing sessions, during which measures of corticospinal excitability and inhibition were obtained using TMS-evoked measures from the first dorsal interosseous (FDI) muscle of the dominant hand. One concussed participant and their matched control were left-handed, and all other participants were right-handed. The hand was placed in a custom-built dynamometer designed to measure force during first finger abduction. Individuals in the CONC group reported to the laboratory within 72 h (2.5 ± 0.7 days) of sustaining their injury and again at 1 week and 2 weeks (±1 day) post-injury. Control participants followed a similar timeline. Measures of height and weight were obtained during the first session.

#### 2.2.1. Electromyography (EMG)

Surface EMG electrodes were placed over the FDI of the dominant hand and recorded all evoked potentials. Prior to electrode placement, the skin was lightly abraded with NuPrep^®^ and cleaned with alcohol to reduce signal impedance. A pre-amplified bipolar, Ag-AgCl electrode (DE-2.1, Delsys Inc., Boston, MA, USA), with an inter-electrode distance of 1 cm was placed over the FDI. This electrode was connected to a portable amplifier (Delsys Bagnoli, Delsys Inc., Boston, MA, USA), which further amplified and band-pass (20–450 Hz) filtered the EMG signal. A ground electrode was secured to the posterior aspect of the distal ulna. The EMG signal was observed on an oscilloscope (TDS 2014C, Techtronix, Beaverton, OR, USA), sampled at 5 kHz with a 16-bit A/D converter (NI USB-6251, National instruments, Austin, TX, USA), and stored on a personal computer for offline analysis using Dasylab software (Data Acquisition System Laboratory, DasyTec, USA Inc., Amherst, NH, USA).

#### 2.2.2. Transcranial Magnetic Stimulation (TMS) Measures

MEPs were elicited from the FDI muscle using TMS (MagStim 200, MagStim Company, Ltd., Whitland, UK) with a flat 70-mm figure-of-eight coil positioned over the optimal site of the contralateral motor cortex, maintaining consistent measures with previous studies [13,16,20,25,36]. With the participant at rest, the optimal site was determined by moving the coil around the head and stimulating at 60% of the stimulator output. The optimal site was defined as the position that consistently yielded the largest response in the FDI, as indicated by the peak-to-peak amplitude of the MEP. Once the optimal site was located, the resting motor threshold (RMT) was determined by reducing the stimulus intensity in a step-wise fashion to find the lowest stimulus intensity required to evoke a response of at least 50 µV in at least 5 out of 10 trials [37,38]. The stimulator was set to 120% of the RMT for the remainder of the study protocol stimulations [15,17,19].

Individuals’ maximal voluntary contraction (MVC) was determined by isometrically abducting the first finger as hard as possible for four to five seconds. Three trials were performed with two minutes of rest in between, and the trial with the greatest force was deemed the MVC. Participants were provided with visual feedback of their force on a computer monitor and asked to contract to 50% of their MVC, while a single-pulse TMS stimulation was delivered to elicit an MEP and CSP. Participants were instructed to maintain the contraction through the stimulation and briefly afterwards. Five trials were performed with ~15–30 s of rest between trials [39,40], obtaining both MEP amplitude and CSP duration from each trial. Corticospinal excitability was assessed through the peak-to-peak amplitude of the MEPs produced. Corticospinal inhibition was assessed through the duration of the CSPs produced. All trials were recorded for offline analysis.

### 2.3. Data Analysis

**Figure 1:**
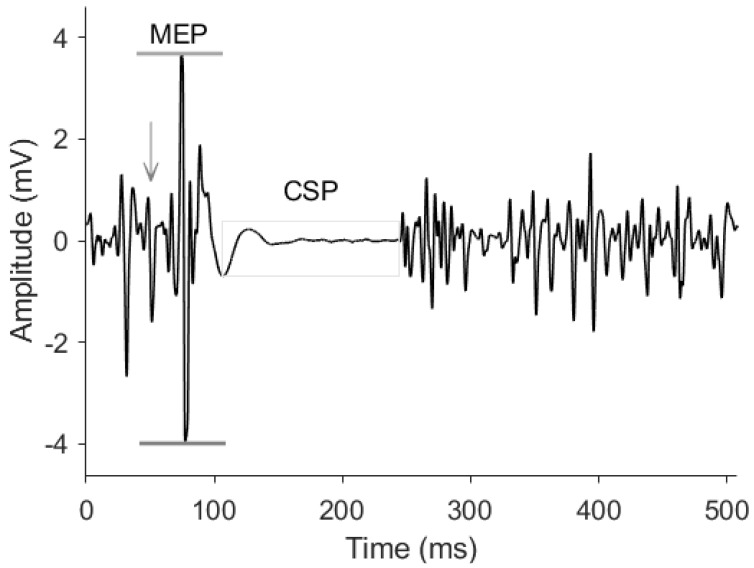
**Sample EMG recording.** Corticospinal excitability was assessed through peak-to-peak amplitude of the MEP (horizontal lines). Corticospinal inhibition was assessed through the CSP duration (shaded area). The arrow indicates the TMS stimulus artifact.

Sample recordings of an MEP and CSP are shown in Figure 1. All trials were analyzed with a custom-written program using MATLAB software (Mathworks Inc, Natick, MA, USA). The peak-to-peak MEP amplitude was determined by marking the onset and offset of the response and calculating the magnitude of the range between the highest and lowest EMG value in the selected period. The silent period was determined as the time between the end of the MEP and the resumption of EMG activity. These points were manually selected by a trained operator, by visual inspection of the raw and rectified signals, which has been shown to be a reliable method of determining EMG onset and offset times [41]. The five trials were then averaged to obtain the MEP amplitude and CSP duration at each time point.

### 2.4. Statistical Analysis

Two-factor (group, sex) analyses of variance (ANOVAs) were used for comparisons of participant characteristics including: age, height, and weight. Two-way repeated measures ANOVAs were used to determine the effect of sex (male vs. female), group (CONT vs. CONC), and time (72 h, 1 week, and 2 weeks), and interaction effects for RMT, MEP amplitude, and CSP duration. Data are presented as mean ± standard deviation (SD) and statistical significance was set at *p* ≤ 0.05. Effect sizes (Cohen’s *d*) for the difference between groups and sexes were also calculated at all time points for each measure to address the magnitude of differences. Effect sizes were interpreted as small (*d* > 0.2), moderate (*d* > 0.5), or large (*d* > 0.8) [42].

Incomplete data sets due to failure to attend all three lab visits for the CONT (*n* = 1 F) and CONC (*n* = 2 F) groups were excluded from analysis, along with those of their matched counterparts. This resulted in a total of 42 participants; 21 in the CONT (9 F) group and 21 in the CONC (9 F) group. There were two outliers (CONT: *n* = 1 M; CONC: *n* = 1 M) in the MEP measurement, as assessed by examination of studentized residuals for values greater than ±3. Therefore, the two outliers and their matched controls were removed, resulting in 19 CONT (10 M) and 19 CONC (10 M) participants for the MEP analysis.

## 3. Results

### 3.1. Participants

Participant characteristics are presented in Table 1. There were no group differences in age, height, or weight (*p* ≥ 0.49), indicating that the groups were well matched. There were significant sex differences in height and weight (*p* < 0.001), as males were generally taller and heavier than females.

### 3.2. Resting Motor Threshold (RMT)

Overall RMT group (CONT and CONC) and sex (male and female) means are presented in Table 2. There was no difference in RMT between groups (*F*(1,38) = 1.482, *p* = 0.23, *d* = 0.25) and sexes (*F*(1,38) = 0.098, *p* = 0.76, *d* = 0.08). There was no effect of time over the two-week testing period (*F*(2,76) = 1.741, *p* = 0.18) and there were no significant interactions (*p* ≥ 0.09).

### 3.3. Motor Evoked Potential (MEP) Amplitude

**Figure 2:**
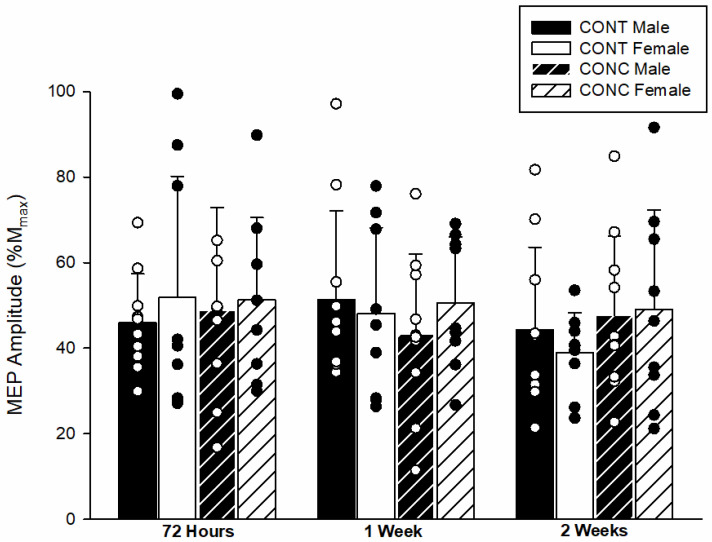
**MEP amplitude across the two-week testing period.** The MEP amplitude was similar between groups (*p* = 0.72) and sexes (*p* = 0.77) across all time points. Circles represent individual data points.

The MEP amplitudes at each time point are shown in Figure 2. There was no difference in MEP amplitude between the CONT and CONC groups (*F*(1,34) = 0.127, *p* = 0.72, *d* = 0.08). There was also no difference between males and females (*F*(1,34) = 0.089, *p* = 0.77, *d* = 0.07) in MEP amplitude. Over the two-week testing period, there was no significant effect of time on MEP amplitude (*F*(2,68) = 0.823, *p* = 0.44) and there were no significant interactions (*p* ≥ 0.43).

### 3.4. Cortical Silent Period (CSP) Duration

**Figure 3:**
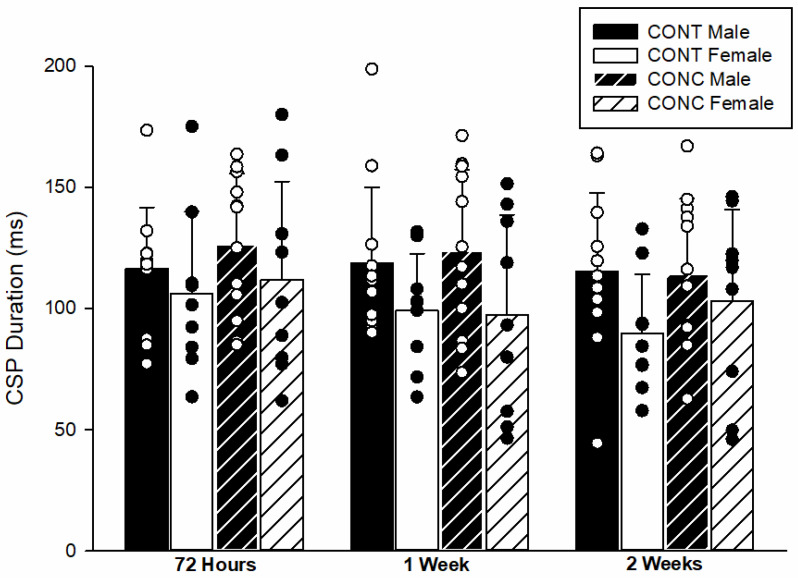
**CSP duration across the two-week testing period.** The CSP duration was similar between groups (*p* = 0.54) across all time points. Overall, males had significantly longer CSP durations than females (*p* = 0.04). Circles represent individual data points.

The CSP durations at each time point are shown in Figure 3. There was no difference in CSP duration between CONT and CONC groups (*F*(1,38) = 0.388, *p* = 0.54, *d* = 0.16). There was a significant sex difference (*F*(1,38) = 4.531, *p* = 0.04, *d* = 0.56), as males had longer CSP durations than females. There was no significant effect of time on CSP duration (*F*(2,76) = 2.091, *p* = 0.13) and no significant interactions (*p* ≥ 0.50).

## 4. Discussion

The purpose of this study was to determine sex-related differences in neurophysiology in the acute and sub-acute post-concussion stages. There were no differences in RMT and MEP amplitude between groups or sexes, and there was no effect of time over the two-week testing period. Therefore, there were no group or sex differences in corticospinal excitability. Although CSP duration was not different between groups, it was significantly longer in males compared to females regardless of group, indicating greater corticospinal inhibition in males. To the authors’ knowledge, this is the first study to examine potential sex-related differences in neurophysiology post-concussion.

### 4.1. Corticospinal Excitability

Our findings of no group differences in corticospinal excitability are consistent with previous studies of concussed individuals within 72 h to 2 weeks post-injury [13,16,20]. A novel contribution of the current study was the demonstration that the lack of difference in corticospinal excitability following concussion was observed in both males and females. Similarity between MEP amplitudes in males and females has been demonstrated previously in healthy individuals [43,44]. However, to the authors’ knowledge, this is the first study to extend these findings to concussed individuals. Although the timeline of the neurometabolic cascade in humans is unknown, reports of similar corticospinal excitability levels between the concussion and control group at 72 h post-injury may be due to the recovery of the excitatory neurotransmitter burst observed only within the first few minutes post-injury in animal models [4]. Therefore, it is possible that 72 h is too long of a post-injury period to capture differences in corticospinal excitability in humans.

### 4.2. Corticospinal Inhibition

There is a general consensus across the literature pertaining to corticospinal inhibition, demonstrating that concussed individuals have longer CSP durations, which remain longer up to 2 months [13,16,20], 9 months [14], 2 years [10], and 30 years post-injury [21]. Alternative TMS-based measures of inhibition, including short- and long-interval corticospinal inhibition, have been used to quantify corticospinal inhibition. These measures generally did not differ between individuals with concussion and healthy controls [14,21,23,45]. The TMS-based measure that most consistently shows a difference between concussed and control groups is CSP, which is frequently of longer duration in concussed individuals [16,17,46,47]. Consistent with previous work, we observed that CSP durations did not change significantly over time. However, in contrast with these previous studies, our results showed that CSP durations were similar between the CONC and CONT groups. Similar to the current findings, a more recent study demonstrated no significant differences in CSP durations between acutely injured individuals and uninjured controls [13]. In the study conducted by Yasen et al. (2020) and the current study, the mean CSP duration was typically longer in the CONC group than the CONT group, but did not reach statistical significance. Together, these studies suggest that while higher levels of inhibition in concussed individuals is a common finding, it is not an inevitable consequence of concussion and should therefore be studied further.

### 4.3. Sex-Related Differences

To our knowledge, the result of significantly longer CSP durations in males compared with females is a novel finding. However, as there was no group-by-sex interaction, these results suggest that sex-based differences in corticospinal inhibition do not likely contribute to previously reported greater PCS scores and longer recovery times in concussed females compared with males [30]. The sex-related differences in recovery time may therefore be related more to other physiological and psychological factors. For example, it has been shown that females have greater interhemispheric communication [48] and greater self-awareness of cognitive deficits [49]. This greater self-awareness and communication may yield greater caution to return to activities and reports of greater symptoms [49].

Pre-menstrual symptoms tend to overlap with concussion symptoms (e.g., feeling upset, anxious, or irritable; headaches; and tiredness or trouble sleeping). As well, studies have demonstrated negative effects on emotional processing in the early follicular (~ days 24–28) and late luteal (~ days 1–8) stages in the menstrual cycle, when hormone levels are declining or low [50,51]. Therefore, varying hormone levels across the menstrual cycle may play a role in the severity and timeline of recovery of post-concussive symptoms, independent of neurophysiological excitability and inhibition. We did not track the stage of the menstrual cycle in this study; however, this may be beneficial in future studies to gain more insight into potential sex-related differences post-concussion.

### 4.4. Limitations

The data presented are from two studies that were conducted at an earlier date. Although we performed all data and statistical analyses presented, the studies were not designed specifically to examine sex-based differences. Human EMG and TMS limitations across multiple testing sessions are inherent in that electrode and/or stimulator placement may not be exactly the same across testing sessions. As well, cortical measures vary within individuals from day to day [52]. Therefore, the natural physiology of the human body may contribute to variability across groups and sexes.

Post-concussion symptom severity scores were obtained from some participants in the dataset, but not all. Therefore, symptom severity scores were not analyzed for the current study. As the number of symptoms reported and recovery of symptoms has been reported to differ between sexes [30,31,33,53], obtaining symptom scores across the testing time would allow a better understanding of the relationship between changes in neurophysiology and changes in symptoms during concussion recovery in males and females. Further, only the dominant hemisphere was tested in this study and it cannot be ruled out that concussion may have a different effect on excitability and inhibition across hemispheres. 

Although five TMS pulses have been shown to produce a reliable MEP amplitude, in a population with greater MEP variability (older adults) [39], a greater number of stimulations may produce more consistent results [54]. We [16,25] and others [40] have used an average of five TMS responses and a single stimulator intensity [15,17,19] in previous work. However, testing with a greater number of trials and range of stimulus intensities may yield different results. Further, when working with acutely concussed individuals, the total duration of the stimulation session should also be considered, in order to not exacerbate symptoms.

Failure to attend every testing session removed three participants and their control counterparts from statistical analysis. This resulted in an unequal number of males and females in the MEP (M = 10; F = 9) and CSP (M = 12; F = 9) measures. In human studies on corticospinal excitability and inhibition post-concussion, there has been a lack of female representation. Similar to this study, the majority of studies have less than 50% female participants [13,16,20,25]. However, these studies did not analyze sex differences. Therefore, more research is required to fully understand the effects of sex on neurophysiology in concussed individuals compared to healthy controls.

## 5. Conclusions

To our knowledge, this is the first study to examine sex differences in neurophysiology in concussed individuals. The lack of significant group-by-sex interactions suggests that differences in corticospinal excitability and inhibition likely do not contribute to the previously documented sex-related differences in symptoms and recovery [30,31,33]. Therefore, further work is necessary to understand the physiology underlying greater symptom scores and longer recovery times in females. An unexpected result in this study was the finding that males had overall greater corticospinal inhibition compared to females, regardless of injury status. This finding highlights the importance of including analyses of sex differences in neurophysiology research.

### Future Directions

An unexpected result in this study was the finding that males had greater corticospinal inhibition compared to females, regardless of injury status. This finding highlights the importance of including analyses of sex differences in neurophysiology research. Therefore, conducting a study on healthy participants with the specific aim of evaluating sex-related differences in corticospinal inhibition may be beneficial to determine if this finding can be replicated in a greater healthy population. Also, it may be beneficial to collect data on menstrual cycle stages upon injury and throughout testing protocols. It has been documented that female hormone levels fluctuate throughout stages, which may affect cognitive and motor processes [50,51,55]. Therefore, it may be beneficial to collect these measures to gain more insight into potential sex-related differences post-concussion.

## Figures and Tables

**Table 1:** Group characteristics.

	Control (*n* = 21; 9 F)		Concussion (*n* = 21; 9 F)	
	*Males*	*Females*	*Males*	*Females*
**Age (years)**	21.75 ± 5.6	20.56 ± 1.1	21.58 ± 5.0	20.11 ± 2.4
Height (cm) *	179.98 ± 5.7	163.20 ± 5.1	179.45 ± 7.7	166.68 ± 8.4
Weight (kg) *	77.98 ± 6.4	64.00 ± 8.2	76.85 ± 13.4	62.57 ± 6.8

Values are presented as mean ± SD. * Males significantly greater than females (*p <* 0.001).

**Table 2:** Overall RMT, MEP, and CSP averages for groups and sexes; Values are presented as mean ± SD. SO = stimulator output.

	Group			Sex		
	*CONT*	*CONC*		*Male*	*Female*	
**RMT (% SO)**	48.25 ± 6.76	46.35 ± 8.01	***p* = 0.23** ***d* = 0.25**	47.56 ± 7.33	46.94 ± 7.65	***p*** **= 0.76** ***d* = 0.08**
**MEP (%M_max_)**	46.8 ± 18.8	48.38 ± 19.36	***p* = 0.72** ***d* = 0.08**	46.91 ± 18.56	48.34 ± 19.69	***p* = 0.77** ***d* = 0.07**
**CSP (ms)**	108.87 ± 29.64	114.0 ± 35.29	***p* = 0.54** ***d* = 0.16**	119.08 ± 29.91	101.24 ± 33.43	***p* = 0.04** ***d* = 0.56**

## Data Availability

Data will be made available upon request.
